# Collectanea Medica, Consisting of Anecdotes, Facts, Extracts, Illustrations, Queries, Suggestions, &c.

**Published:** 1818-07

**Authors:** 


### COLLECTANEA MEDICA,

CONSISTING OF

### ANECDOTES, FACTS, EXTRACTS, ILLUSTRATIONS, QUERIES, SUGGESTIONS, &c.

RELATING TO THE

*History or the Art of Medicine, and the Auxiliary Sciences.*

Quicquid agunt medici,
nostri farrago libelli.

### *Oration on the Death of Dr. Woodville, by* Mr. Highmore.

[One of our former Numbers contained a communication expressive of the uncourtly manner in which Mr. French treats those who he supposes may differ from him. The following are in vindication of a learned and respectable name no longer able to protect itself.]

There is not, perhaps, any reflection which affords more soothing consolation to concern at the loss of relatives or friends, than that which dwells on the remembrance of their merits, and recapitulates the history of their virtues;—whatsoever may have been the station of any individual, his peculiar profession, or the general course of his occupations, these either furnish unequivocal testimonies to his fame, and transmit his character with sympathy and esteem to his nearest relatives, or hand it with eulogy and renown to the listening admiration of a remote posterity. We fix upon the generous qualities of his heart, or upon the enlightened liberality of his mind, as a centre from whence his public action or his private worth emanate as radii, which expand to their distant circumference as the congenial spirit of veneration and respect bears testimony to their justice, and magnify, but not exaggerate, their truth.

Which of us, my respected friends, have not thus sympathized with the tears of sorrow, and thereby mitigated the mournful agonies of distress? Who is there amongst all the sons of Adam, who has not borne the sighs of grief, and wept with those that weep?

We have here no common cause for our concern: the tribute of our tears is the last that we can offer to the merits of the man! the tribute of our respect is due to his public character—the tribute of veneration and applause is the debt we owe to his fame!

It is with affecting delight we contemplate the merit of our departed friend, and review his eminent services since his introduction to the Small-pox Hospital; and they seem to have reflected back the honour which they cast upon each other. As patrons of this house of mercy, you have revered his skill, and duly appreciated his exertions in its cause: you have seen the energ es of his mind devoted to its extension, and the fruits of his beneficial improvements have transmitted its name to remotest climes.

His qualifications as a physician, and his merits as a man, were considered fourteen years ago by the ample patronage he received at his first introduction to the office which his disease has now vacated. His studies and researches in the science of medicine were then called forth into new action, for they were made subservient to the cause in which he had thus engaged, and formed a considerable part of his general practice:—his mind willingly devoted itself to the fulfilment of his engagement, by not only conducting its medical department, but by also taking the supervision and direction of its domestic household;—the regulations which his care and vigilance have introduced remain as monuments of his skill, and as testimonies of his paternal regard.

Five years after his introduction to this office, he began the compilation, of which only the first part has appeared, of a *History of Inoculation;* which ought to constitute a leading feature of his literary labours, as it proves how deeply the design was impressed upon his mind, of fulfilling the extent of his duty by the most attentive investigation, wherein nothing might be left unexplored which could contribute to elucidate or promote the objects of his situation.

Amongst his literary labours, which afforded no small assistance to his profession, and reputation to himself, and which offers a further testimony how deeply every part of the medical science was within the scope of his attention, was his work on *Medical Botany*—an accurate delineation of the science of plants, and a useful and pleasing inquiry into the vegetable kingdom: here he explored the forms and natures of the—

> " Living herbs, beyond the powers
> " Of botanists to number up their tribes."—*Thomson.*

Whilst the mind of Dr. Woodville was thus ardently engaged in studies which enlarged his own sphere of knowledge, and secured to him the well-earned honours of professional reputation, it will excite no surprise to find him zealously engaged in the discovery and adoption of *Vaccine Inoculation.* A discovery so fortunate for mankind, and which so immediately affected the advancement of this institution, could not fail to attract his vigilance, and to press for his mature investigation. As its course proceeded, he was enabled, from his peculiar office, and was urged from his peculiar benevolence, to communicate many essential observations and improvements, which tended to methodise the discovery, and to push its new-born light upon the world, to remove the suspicions of fear, and to promote and mature the blessings of security.

After the minutest experiment, and the most unequivocal testimonies of its success, it was to our departed friend that this institution claims the honour of its introduction into general practice in the metropolis; and, as one of the branches of the establishment, to have been instrumental, superadded to the subjects of its former fame, in conveying comfort and security to more than 17,000 persons, during the last six years; a number which, in addition to those which have received the same benefit from other societies, and from the liberal exertions of other medical men, will live to teach their children, and their children's children, to bless the name of Woodville, when they bless the name of Jenner.

What best characterized his medical genius, was the solidity of his conceptions, the caution of his measures, and the prudence which prevented their adoption until he had ascertained their final effects: it may without exaggeration be affirmed, that there are few men who present us with such inestimable lessons in the study of public utility.

But his exertions and his fame were neither limited by the narrow circuit of these walls, which now hold his silent remains, nor even by the expanded boundaries of the metropolis, nor yet by the shores which girt our united kingdom; his reputation stretched to many or most of the states of Europe, to the provinces of America, and to the colonial establishments in the eastern and western world:—from all these parts his various correspondences, and particularly the earnest desire with which his presence was solicited at Paris, during the late consulship, and granted by the British government, prove the ardour with which his opinions were sought and esteemed; to these he freely imparted the result of his judgment and the correct information of his practice: and, if such an intercourse diffused the character of his own talents, it also carried along with it the fame of this national institution to the remote corners of the globe.

Glorious must have been the inward satisfaction of his own benevolence, that he was thus guiding, under the blessing of Divine Providence, the great purposes of the institution whose principle he superintended, by not only extending relief to affliction itself, but by combining the most effectual measures for protection against it, with the most active co-operation with other societies, for its final extermination.

It is thus, through these men, that the formidable hydra, whose venom was the terror of our ancestors, has in our times been devoted to its ruin—it is thus that the monster of Peloponnesus no longer scatters devastation from every wound; these men have the modern glory of neutralizing her virus, and, mingling their labours with those of Hercules himself, they will commit her story to the records of former times; while the dark shade which once obscured the happiness of human life shall be dispelled by a noontide radiance, presenting to the admiring gaze of posterity the characters of Jenner and of Woodville, written with a sun-beam.

Yet, notwithstanding these extensive powers, this public usefulness, this thirst for medical knowledge, this almost universal reputation, the modest diffidence of our departed friend shrunk from the blazonry of fame, and almost forbade its voice;—the silent consciousness of extensive merit rather led him to rejoice in the effect, than to condescend in self-flattery to the cause;—he rejoiced to see the prosperity of his plans, but the delicate humility of his heart ascribed to a sublimer source the dictation and the glory.

If the esteem and approbation of a few cordial friends were the limits of his ambition—if he preferred not to barter a jewel of so inestimable a price for the transitory breath of popular fame, it is for those friends to dwell upon his virtues; and now, that his humility does not repress their zeal to furnish the triumph of applause, they may exemplify his merits, which the loud herald of renown has not rendered common; they may dwell upon his character and his exertions, which the trump of eulogy can never tarnish by exaggeration.

As a public officer of this house, his liberal and active attention to all its departments was not limited by the rigid letter of his duty, but rather extended by the benevolence of his heart. His unwearied regard to the comfort and safety of those committed to his care, his gentle treatment of the afflicted, and his encouragement of the convalescent, procured him their respect and gratitude,—and his punctual regularity, and affectionate interest, in the welfare of the household over which he was the deputed guardian and general visitor, has left an example for his successor until the house itself shall be removed:—happy, thrice happy, if that successor shall tread his path, and transfer to himself the remnant of his fame.

Endowed with urbanity of manners, warmed with the zeal of friendship, and ennobled with the self-possession of that *mens sibi conscia recti* which dignifies and elevates the human heart, which upholds in sorrow, and gives equanimity in the dangers of prosperity, we may reflect with satisfaction that our friend is departed but a little while before some of us; and, if we contemplate his virtues, and emulate his example, we may hope to follow and to meet him where tears and sorrows shall be wiped away.

### *Biography of Dr. Woodville.*

—From the public Grammar School of our native town (Cockermouth), Woodville, a young quaker, had just been removed, and placed behind the counter of an apothecary, on the side of the street directly opposite my father's house; when, having learnt to read and spell, and say the *long* assembly's catechism by rote, I was sent to the same school, at six years of age, to learn the more easily comprehensible rules of Lily on a dead language. He had several coeval apprentices in the town, of the same profession, and soon, I remember well, he was not too conceited to appeal to his little neighbour school-boy, in disputing with his brother Esculapianinos on the rules of grammar. But, is not this, *à la Moore*, bringing forward the puff-ball? Let us pass on then to the man's estate of the author of the Medical Botany. He had graduated, travelled on the continent, and returned to his native home; when, residing at his mother's, at a neighbouring village, after midnight, the rest of the family being gone to bed, he heard the tread and low-toned voices of men hovering about the premises, who seemed to take their stand by the window of the room in which he was sitting. On his calling out to them, they made no answer; but, on his going to the kitchen with his light, to see if all was safe there, (a fowling-piece loaded with shot in his hand,) one of them coming up to the window, which looked into a private yard, he renewed his call to them, when, moving forwards and backwards without making any answer, they appeared evidently reconnoitering. Coming again close up to the window, he called out, "If you don't be gone, I'll shoot you;" and, on presenting the piece, he actually broke the window with the muzzle of it, and, in the agitation of the moment, involuntarily pulled the trigger, when a poor innocent youth immediately fell: he was related to a maid-servant in the house, and had accompanied his friend, another young man, a soldier, their first cousin, by appointment, to take his leave of her previous to his setting out for the army the next morning. These worthy young men had taken a cheerful glass with some friends in the town, and not been punctual to the appointment. They were so much past the time, that the young woman had given them up, and gone to bed.

Dr. Thornton had stated, and printed, that the melancholy event had happened on a clear moon-light night, as if Woodville might have seen and known who the seeming assailants of his house were at the time. But, on my informing him that, at the very time, a party of us Cumberland youths, after a festive supper, were beating up with terriers the thickets and bushes of the hills and dales of that country, in search of those animals so destructive to the hen-roost, and that, though a frosty, it was a cloudy or misty, night, with snow enough on the ground, (but only at a distance from the town or village,) to light us through our nocturnal expedition,—he cancelled the sheet, and afterwards thanked me for preserving him from obtaining the name of a detracter of the dead.

Already, in the vindication of an old friend, I feel that *Jamque opus exegi* for which the liberal of the profession will give me credit. It will make them tolerate, perhaps, with some indulgence, the egotism of a man who, tossed about the world rather *eccentrically* nearly half a century since he first left his native hearth, is now put upon the defence of his professional character—that is, of his bread. I shall have afforded them a gratification scarcely in the power of any other individual—the gratification of seeing rescued the good name of a late estimable character from the misrepresentations of the calumniator.

I now conclude on this departed character, "*mourant en Socrate*," as I heard Lafisse well express it, at a public sitting of the *Societé de Medicine de Paris*, in his *Eloge de Vicq d'Azyr*, in alluding to the patient sufferings of Baillie, Mayor of Paris, at the time of his death, yet without withholding the expression of the wish—that, when the time shall arrive when we may wish to get rid even of the faintest recollections of the feuds and animosities, wherein we reciprocally attempted the discomfiture or harassment of the opponent, "or even the silver cord be loosed, or the golden bowl be broken, or the pitcher be broken at the fountain, or the wheel broken at the cistern," when the dust shall return to the earth—the wish that our latter end may be like his.

I conclude with presenting the information I have derived directly from the officers of the Small-Pox and Inoculation Hospital.

Dr. Adams, the successor of Dr. Woodville, as physician to the Small-pox and Inoculation Hospital, informs me, that, in conversing with him a little before his death, he complained to him of medical men addressing him as they would their own patients, even expressing hopes of his recovery, whilst they must know that he could not be insensible of the impossibility of such an event. A few days before his decease, his friend Coleman, professor at the Veterinary College, playing at chess with him at the hospital, the arrival of Mr. Greatrex, an undertaker, was announced. On his entering, the doctor requested him to sit down, saying, the game was nearly finished:—"Mr. Greatrex, there was a misunderstanding between you and me in my settling with you on leaving Ely-place, and I am desirous of showing you that I am at peace with you, as I hope to die in peace with all men. I am just on the point of departing, have but very few days to live, and wish to arrange with you the order of my funeral." The astonished undertaker had never before been so addressed; hoped the doctor was quite mistaken, that he might yet live many years.—"Many of my friends, Mr. Greatrex, address me in this way; and, were I young, I might wish a restoration to health, but the system is done—a return to health and vigour is impossible. Having been brought up a quaker, I wish to be buried amongst them, and in their plain way, to avoid every unnecessary expense. You will, therefore, now consider the amount of the necessary disbursements for the occasion, and I will discharge them. It will save some trouble to my executors."

---

### *Sketch of the Life and Character of the late* Benjamin Rush, M.D. L.L.D. Professor of the Institutes and Practice of Medicine in the University of Pennsylvania, &c.

(From the American Medical and Philosophical Register.)

Benjamin Rush was born near Bristol, in the state of Pennsylvania, on the 5th of January, 1745. His ancestors belonged to the society of Quakers, and were of the number of those who followed the celebrated William Penn to Pennsylvania, in the year 1683: his grandfather, James Rush, resided on his estate near Philadelphia, and died in the year 1727. His son inherited both his farm and his trade, which was that of a gunsmith. He died while Benjamin was yet young. His widow, a most excellent woman, upon whom the education of young Rush was devolved, placed him, at an early age, under the direction of the late Rev. Samuel Finley, afterwards better known as the president of Princeton College, New Jersey. He was an able scholar and faithful teacher: being related to Mrs. Rush, he may be supposed to have paid great attention to the improvement of his young pupil. But whatever may have been the assiduity with which his education was directed by his preceptor, he possessed an ardent desire for knowledge, and was most unwearied in the pursuit of it.

From the academy of Dr. Finley he was removed to the college of Princeton, where he finished his classical education, and was admitted to the degree of A.B. in 1760, when he had not yet completed his sixteenth year. He was now left to choose a profession, and seems to have fully known his own character, and to have formed a proper estimate of his talents, and, by applying them to the science and practice of medicine, to have been desirous of doing all possible good to the family of mankind. That he was directed by these motives, may be inferred from his own opinion of the utility of medicine. "So great," says he, "are the blessings which mankind derive from it, that, if every other argument failed to prove the administration of a Providence in human affairs, the profession of medicine would be fully sufficient for that purpose."

He accordingly, soon after leaving college, placed himself under the care of the late Dr. John Redman, of Philadelphia, a gentleman who had deservedly obtained an extensive share of professional business. With Dr. Redman young Rush continued some time, zealously engaged in the acquisition of the several branches of medicine. But, feeling his thirst for knowledge being rather excited than gratified, with what he had learned from his preceptor, he formed the resolution of going abroad, in order to avail himself of those advantages which were not within his reach in this country. The university of Edinburgh, at that time, was at the zenith of its reputation, and justly boasted of its able professors, among whom were the elder Munro, the elder Gregory, Dr. Cullen, and Dr. Black. Thither Rush repaired, and was graduated M.D. in 1768, after having performed the usual collegiate duties with much honour, and published his inaugural dissertation *De Concoctione Ciborum in Ventriculo.* In this performance he candidly acknowledged himself indebted, for many of the opinions which he advanced, to his distinguished teacher, Dr. Cullen.

About the period of Dr. Rush's return to his native country, the first attempt was made in Philadelphia for the organization of a medical school. Lectures on anatomy and surgery had indeed been delivered, in that city, in 1763 and 1764, to a small class of pupils, by the late Dr. William Shippen, who, two years previous, had returned from Europe, where he had completed his education under the direction of the celebrated Dr. William Hunter; and, in 1765, Dr. John Morgan also gave instruction on the institutes of medicine and the practice of physic. Three years after this, the venerable Dr. Kuhn, who had been a pupil of the illustrious Linnæus, and had preceded Dr. Rush in his medical honours at Edinburgh only one year, was made the Professor of Botany and the Materia Medica. To this list of teachers, Dr. Rush himself was added as Professor of Chemistry, immediately upon his arrival from England in 1769. Such was the first organization of the medical college of Philadelphia.

That Dr. Rush had, in an eminent degree, the qualifications of a teacher, and that he discharged with exemplary fidelity the important duties belonging to the elevated station to which he was chosen, the popularity attending his lectures, the yearly increase in the number of his hearers, and the unexampled growth of the college with which he was connected, bear ample testimony. Shortly after this period, he was elected a fellow, and also one of the curators of the American Philosophical Society. While Dr. Rush was thus engaged in the active pursuits of his profession, the dispute of the then American colonies with Great Britain arose. Considering the claims of the British government unjust, he entered with warmth into the defence of the rights of his countrymen. His talents were already well known, and the fullest confidence was placed in his integrity and patriotism. The crisis demanded his services. In the year 1776 he was chosen a member of congress for the state of Pennsylvania, and, on the 4th of July, with eight other delegates from that state, he signed the instrument of independence. Upon the 11th day of April, 1777, he was appointed surgeon-general of the military hospital in the middle department. This office was of short duration: he indeed fulfilled the duties of his appointment with all the promptitude suitable to the exigency of the times; but, as he had more particularly directed his studies to the practice of physic, and deemed himself better prepared to watch over the concerns of the medical department, he gave a decided preference to a station of that kind. His wishes were soon granted; for upon the 1st of July, 1777, he was created physician-general of the hospital, in the middle department, in the room of Dr. Jones.

On the 6th of February ensuing, Dr. Rush resigned the station of physician-general, and Dr. William Brown was appointed in his place. We shall here forbear any remarks upon Dr. Rush's resignation, nor shall we offer any conjectures as to the causes which led to it. We are certain he did not quit the public service on account of any difficulties incident to the office, or from any want of inclination to promote, by every honourable means, the cause of his country. Serious disputes had arisen among the heads of the medical staff, and there existed many heavy charges of impropriety of conduct on the part of the managers of the hospital stores, which eventually terminated in an investigation by a court of inquiry. The character of Dr. Rush remained unsullied, and, being determined to remain free, even from the suspicion of mal-conduct, he totally withdrew himself from the hospital department.

[On the subject of Dr. Rush's resignation, the American biographer seems to dwell too shortly. When an upright man is found conscientiously attentive to the duties of his office, his less principled colleagues will call him troublesome, and accuse him of interested motives. First, he bears patiently, but indignant; at the second, hastily resigns, expecting thus to shew the injustice of his accusers. By this fatal deed he accomplishes all that his enemies wish; they have now an open field before them, are all probably, more or less, defaulters, and their time and thoughts are occupied in aggrandizing themselves and deceiving the public, instead of their official duties. The only apology for Dr. Rush is in the purity of his own heart and ignorance of the depravity of others. —English Edit.]

Dr. Rush, however, still continued to take an active part in the politics of the state to which he belonged. The original government of Pennsylvania is known to have been perfectly unique in its form, and the constant source of incalculable mischief. The house of representatives, chosen annually by the people, and on which there was no check, was the sole legislative power: and each succeeding assembly often made it their business to undo all that their predecessors had done. This kind of government was justly reprobated by Dr. Rush, and the necessity and wisdom of a reformation in it was too apparent not to be attempted. Dr. Rush, and many other distinguished abettors of the cause, had, soon after, the satisfaction of seeing a new form of government established in Pennsylvania, by a general convention of the people.

He who has acquired a high reputation in pursuits which do not belong to his profession, is doubly obligated to establish a character as a successful cultivator of that art or science to which his primary attention may have been directed. Of this circumstance Dr. Rush was fully aware: he was already acknowledged an able teacher and practitioner of medicine; but he had also devoted a considerable portion of his time to politics and legislation. But, after the fortunate issue of the revolution, he formed the resolution of retiring from political life, and of devoting the remainder of his days, with increased ardour, to his profession. He was still further induced to this resolution, from the consideration of the state of medicine in his native country at that time, which, it is scarcely necessary to remark, was in a very low condition. Happy for the interests of humanity, that he so early formed such a resolution, and that he was so steady, uniform, and indefatigable in the accomplishment of it.

During the long and brilliant career of Dr. Rush's life, from this time to its termination, he may be considered as exclusively occupied in duties pertaining to his profession, and not unlike another Howard, in "surveying the mansions of sorrow and pain," and in mitigating and removing the distresses of all within his power.

His biography, therefore, like that of most other scientific men, consists chiefly in a history of his professional labours. How numerous and important his services, as an author, have been, will be readily seen from a brief detail of his writings, which we shall attempt to give, as nearly as practicable, in chronological order.

The first fruits of his professional labours, as an author, was an account of the effects of the Stramonium or thorn-apple: this appeared in the year 1770, and was published in the Transactions of the American Philosophical Society, vol. 1st. The same year he addressed a letter, on the Usefulness of Wort in ill-conditioned Ulcers, to his friend Dr. Huck, of London, which was published in the Medical Observations and Inquiries of London, vol. 4th. In 1774 he read, before the Philosophical Society, his interesting Inquiry into the Natural History of Medicine among the Indians of North America, which formed the subject of an anniversary oration. He this year again addressed another letter to Dr. Huck, containing some Remarks on Bilious Fevers, which was printed in the London Medical Observations and Inquiries, vol. 5th. To this succeeded his Account of the Influence of the Military and Political Events of the American Revolution upon the Human Body, and Observations on the Diseases of the Military Hospitals of the United States, which his situation in the army eminently qualified him to make. In 1785, he offered to the Philosophical Society of Philadelphia an Inquiry into the cause of the Increase of Bilious and Intermitting Fevers in Pennsylvania; published in their Transactions, vol. 2d; and soon after, in quick succession, appeared Observations on Tetanus, an Inquiry into the Influence of Physical Causes upon the Moral Faculty, Remarks on the Effects of Ardent Spirits upon the Body and Mind, and his Inquiry into the Causes and Cure of the Pulmonary Consumption. About this time also, appeared his paper, entitled Information to Europeans disposed to migrate to the United States, in a letter to a friend in Great Britain; a subject which had already occupied the attention of Dr. Franklin, but which Dr. Rush considered still further deserving notice, on account of the important changes which the United States had lately undergone. To this paper followed his Observations on the Population of Pennsylvania, Observations on Tobacco, and his Essay on the Study of the Latin and Greek Languages, which was first published in the American Museum of Philadelphia. This last-mentioned paper, which has been the fertile topic of much animadversion, was, with several other essays of Dr. Rush, and his Eulogiums on Dr. Cullen and the illustrious Rittenhouse, the former delivered in 1790, the latter in 1796, embodied in an octavo volume, entitled, Essays, Literary, Moral, and Philosophical, and published in 1798.

In 1791, the medical colleges of Philadelphia, which, on account of certain legislative proceedings since the year 1788, had existed as two distinct establishments, became united under the name of the University of Pennsylvania; and Dr. Rush was appointed to the chair of the professorship of the Institutes of Medicine and

Clinical Practice. He now gave to the public his Lectures upon the Cause of Animal Life. The same year he presented the Philosophical Society his Account of the Sugar Maple Tree of the United States, which was published in their Transactions, volume 3d.; and, in 1792, Observations, intended to favour a supposition that the Black Colour of the Negro is derived from Leprosy, published in their Transactions, volume 4th.

The year 1793 is memorable in the medical annals of the United States, from the great mortality occasioned by the yellow-fever which prevailed in the city of Philadelphia. The history of that epidemic, which was published by Dr. Rush in 1794, cannot be too highly valued, both on account of his minute and accurate description of the disease, and the many important facts he has recorded in relation to it. It was comprised in one volume octavo, and has undergone several editions, and been extensively circulated in the Spanish and in the French language. About this period, also, he offered to the medical world his Observations on the Symptoms and Cure of Dropsy in general, and on Hydrocephalus Internus; an Account of the Influenza, as it appeared in Philadelphia in 1789, 1790, and 1791; and, Observations on the State of the Body and Mind in Old Age. In 1797, came out his Observations on the Nature and Cure of Gout, and on Hydrophobia; an Inquiry into the Cause and Cure of the Cholera Infantum; Observations on Cynanche Trachealis, &c.

It is proper to state, as connected with the literary labours of Dr. Rush, that, in 1788, many of his medical papers were collected together, and that he offered them to the public under the title of Medical Inquiries and Observations, volume first. These he from time to time continued, embracing most of the writings above enumerated, besides Observations on the Climate of Pennsylvania, and some others, until a fifth was completed in 1798. In 1801, he added to his character, as a writer, by the publication of Six Introductory Lectures to a Course of Lectures upon the Institutes and Practice of Medicine, delivered in the University of Pennsylvania. In 1804, a new and corrected edition of his Medical Inquiries, &c. was printed in four volumes octavo. In 1806, he also published a second edition of his Essays. In 1809, such was the demand for the Medical Inquiries and Observations, he again revised and enlarged the work throughout, and enriched the medical profession with a third edition. In this edition he continued his several histories of the yellow-fever, as it prevailed in Philadelphia, from 1793 to 1809. It also contained a Defence of Blood-letting, as a Remedy for certain Diseases; a View of the comparative State of Medicine in Philadelphia between the years 1760 and 1766, and the year 1809; an Inquiry into the various Sources of the usual Forms of Summer and Autumnal Diseases in the United States, and the Means of preventing them; and the Recantation of his Opinion of the Contagious Nature of the Yellow-fever.

He now formed the idea of selecting some of the best practical works for republication in America; and, in order to render them more useful, of adding to them such notes as might the better adapt them to the diseases of his own country. His editions of Sydenham and of Cleghorn were published in 1809, and in 1810 appeared those of Pringle and Hillary. In 1811 appeared a volume of Introductory Lectures, containing those he had formerly published, with ten others, delivered at different years before his class, and also two upon the Pleasures of the Senses and of the Mind. His work upon the Diseases of the Mind, which had long and ardently been looked for, was next added to his writings. It appeared towards the close of 1812, in one volume, octavo. The last effort of his pen was a Letter on Hydrophobia, containing additional reasons in support of the theory he had formerly advanced, as to the seat of the disease being chiefly in the blood-vessels. It was addressed to Dr. Hosack, and written not many days before his fatal illness.

While thus assiduously engaged in enriching medical science with the valuable fruits of his long and extensive experience, and in the active discharge of the practical duties of his profession, he was, on the evening of the 13th of April, seized with symptoms of general febrile irritation, which were soon accompanied with considerable pain in his chest. His constitution was naturally delicate; he had acquired, from previous illness, a predisposition to an affection of his lungs. He lost a moderate quantity of blood, by which he felt himself considerably relieved. But his strength was not sufficient to overcome the severity of his complaint: the beneficial effects resulting from the most skilful treatment were but of a temporary duration. His disease rapidly assumed a typhus* character, attended with great stupor and a disinclination to conversation. In other respects, however, he retained his faculties, and the perfect consciousness of his approaching dissolution. On Monday evening ensuing, after a short illness of five days, and in the 69th year of his age, he ended his truly valuable and exemplary life. His death was the subject of universal lamentation, and he was followed to the grave by thousands, who assembled to bear testimony of his excellence.

In January, 1776, he married Miss Julia Stockton, daughter of the Honourable Judge Stockton, of New Jersey, a lady of an excellent understanding, and whose amiable disposition and cultivated mind eminently qualified her as the companion of Dr. Rush. Thirteen children were the fruits of their marriage, nine of whom still survive. Two of these are chosen to offices of high respectability in the general government of the United States.

It were no easy task to do adequate justice to the great talents, the useful labours, and the exemplary character of Dr. Rush. From the preceding sketch, it is presumed, some idea may be

* On the delicate events attending Dr. Rush's illness, we shall only refer our readers to Mr. Pettigrew's Memoir of Dr. Lettsom, and to the extracts in our Journal.—Eng. Edit.

formed of his incessant devotedness to the improvement of that profession of which he was so bright an ornament. His merits, as a practitioner, are too well known to need particular notice: he was fully aware of the great responsibility attached to the medical character, and uniformly evinced the deepest solicitude for the recovery of his patient. His kindness and liberality in imparting aid to those from whom no remuneration was ever to be expected was unbounded, and arose from the generous impulse of his nature, the cordial concern he felt in whatever affected the interests of his fellow creatures. His mind was of a superior order: to a perception naturally ready and acute, he united a discriminating judgment, a retentive memory, which was greatly improved by habits of close attention, a brilliant imagination, and a highly cultivated taste. He possessed a comprehensive understanding; his knowledge was varied and profound, and he eminently excelled in the several departments of his profession. In his assiduity and perseverance in the acquisition of knowledge he had no superior, and few equals. Accustomed to constant and regular exercise, his intellectual powers acquired additional vigour from employment. Notwithstanding the great fatigue he had to undergo in the discharge of the practical duties of a laborious profession, and the constant interruptions to which he was exposed, when engaged in his pursuits as an author, he never for a moment abated of his ardour in the cause of science. His habits of punctuality to every kind of business in which he was employed, added to a judicious arrangement of time for his multifarious occupations, secured to him sufficient leisure for the publication of those works which have given such celebrity to his name.

His writings claim our attention, both on account of their extent and their variety. Instead of being a mere collator of the opinions of others, he was constantly making discoveries and improvements of his own; and, from the results of his individual experience and observation, added more facts to the science of medicine than all who had preceded him in his native country. His description of diseases, for minuteness and accuracy of detail, cannot be exceeded, and may safely be regarded as models of their kind. In the treatment of gout, dropsy, consumption of the lungs, and the diseases of old age, he enlarged our views of the animal economy, and threw more light upon the peculiar character of these afflicting disorders, than is to be derived from the investigations of any other writer. His volume on the diseases of the mind, in as far as it shows the infinitely varied forms which those ideas exhibit, is a storehouse of instruction. Had his labours been limited to these subjects alone, his character would deservedly have been cherished by future ages. His reputation, however, will permanently depend upon his several histories of the epidemics of the United States, which have rendered his name familiar wherever medical science is cultivated, and will hereafter cause to be inscribed upon the same imperishable column that bears testimony to the merits of Sydenham and Boerhaave, the illustrious name of Benjamin Rush. The respect and consideration which his publication procured for him among his contemporaries, was such, that the highest honours were accumulated upon him in different parts of Europe, as well as in his own country, and he was admitted a member of many of the most distinguished literary and philosophical associations.

There are other qualities which still more entitled Dr. Rush to our respect and esteem. In private life, his disposition and deportment were in the highest degree exemplary. Admired and courted for his intellectual endowments, he riveted to him the affections of all who enjoyed the pleasure of an intimate acquaintance. The affability of his manners, the amiableness of his temper, and the benevolence of his character, were ever conspicuous. He was ardent in his friendships and forgiving in his resentments; and yet, entertaining a due regard for himself, and a high sense of honour, he possessed a manly independence of spirit, which disdained every thing mean and servile. He had an extraordinary command of language, and always imparted his thoughts in a peculiarly impressive and eloquent manner. Those who had the happiness to experience the delights of his conversation, will long recollect, with pleasure, his unassuming modesty, and the rich stores of knowledge he poured forth on the most instructive topics. Even when his opinions were solicited, they were given, not as the dictates or admonitions of a superior, but as the kind advice of a friend and equal. He never evinced any of that haughtiness and affectation of importance, which sometimes attaches to men of eminence, and which so materially lessens the pleasures and comforts of social life.

He was a believer in Christianity from an examination of its principles, and the deepest conviction. The purity of its doctrines and the excellence of its precepts was a frequent topic of his conversation: its practical influence upon his conduct through life he often acknowledged, and cherished with a fervent hope the animating prospects which it affords. His writings, in numerous places, bear testimony to his Christian virtues; and, in a manuscript letter, written a short time previous to his fatal illness, and now before the writer of this imperfect sketch, he candidly declares, that he had "acquired and received nothing from the world which he so highly prized as the religious principles he received from his parents." It is peculiarly gratifying to observe a man so distinguished in a profession in which, by the illiberal, religious scepticism is supposed to abound, directing his talents to the maintenance of genuine piety, and the enforcing of Christian virtue. To inculcate those principles which flow from the source of all truth and purity, and to impart them as a legacy to his children, was an object dear to his heart, and which he never failed to promote by constant exhortation, and the powerful influence of his own example.

